# Reemerging Rice Orange Leaf Phytoplasma with Varying Symptoms Expressions and Its Transmission by a New Leafhopper Vector—*Nephotettix virescens* Distant

**DOI:** 10.3390/pathogens9120990

**Published:** 2020-11-26

**Authors:** Gilda B. Jonson, Jerlie M. Matres, Socheath Ong, Toshiharu Tanaka, Il-Ryong Choi, Sotaro Chiba

**Affiliations:** 1 Rice Breeding Platform, International Rice Research Institute, Los Baños, Laguna 4031, Philippines; G.Jonson@irri.org (G.B.J.); J.Matres@irri.org (J.M.M.); I.Choi@irri.org (I.-R.C.); 2 Department of Crop Protection, Faculty of Agronomy, Royal University of Agriculture, Ministry of Agriculture, Forestry and Fisheries, Chamkar Duang, Dangkor District, Phnom Penh 370, Cambodia; osocheath@rua.edu.kh; 3 Nagoya University Asian Satellite Campuses Institute—Cambodian Campus, Royal University of Agriculture, Phnom Penh 2696, Cambodia; 4 Plant Pathology Lab., Graduate School of Bioagricultural Sciences, Nagoya University, Furo-cho, Chikusa-ku, Nagoya 464-8601, Japan; totanaka@agr.nagoya-u.ac.jp

**Keywords:** rice orange leaf phytoplasma, *Candidatus* phytoplasma asteris 16SrI-B subgroup, reemergence, transmission

## Abstract

Rice orange leaf phytoplasma (ROLP) belongs to the “*Candidatus* Phytoplasma asteris” 16SrI-B subgroup, which is solely transmitted by the zigzag-striped leafhopper (*Recilia dorsalis* Motchulsky) and the green leafhopper (*Nephotettix cincticeps* Uhler) (*Hemiptera: Cicadellidae*). Recently, rice plants showing orange leaf discoloration have become ubiquitous in several paddies of two provinces in the Philippines. In total of 98 symptomatic rice plants, 82% (Laguna) and 95% (Mindanao) were ROLP-positive by nested PCR detection. These plants showed more varying symptoms than previously reported. The vector insect *R. dorsalis* was scarcely present but green paddy leafhopper, *N. virescens* Distant (*Hemiptera: Cicadellidae*), was commonly observed in the paddies, thus the ability of *N. virescens* to transmit ROLP was thoroughly investigated. Newly emerged adult *N. virescens*, which fed on ROLD-source rice plants, were used to inoculate a susceptible rice seedling and was serially transferred into a new healthy seedling. Resultant positive transmission rates varied from 5.1% to 17.8%. The transmission ability of the insects was generally decreased over time. These findings suggest that *N. virescens* is an alternative vector of ROLP in the Philippines. Altogether, this study highlighted the increasing importance of ROLD-reemergence in Southeast and East Asia and proved the need for careful management of this alternative vector insect.

## 1. Introduction

Rice orange leaf disease (ROLD) was first reported in the Philippines in 1960 [1]. The causal agent—rice orange leaf phytoplasma (ROLP)—was identified in 1987 based on insect transmission tests and by electron microscopy observations [2]. Another phytoplasma species, which causes a disease in rice called rice yellow dwarf phytoplasma (RYDP) has been identified [3]. Phytoplasma belongs to a family of bacteria that is unculturable in vitro. Taxonomically, phytoplasma belongs to the class Mollicutes, and has been classified within the genus “*Candidatus* Phytoplasma” based on 16S rDNA sequence analysis [4]. Presently, molecular analysis of conserved genes, particularly the 16S rRNA gene, is used for the detection, identification, and classification of phytoplasmas by conventional and nested polymerase chain reaction (PCR) assays [5,6,7]. The ROLP isolates from the Philippines [8] and India [9] were shown to belong to the 16SrI “*Candidatus* Phytoplasma asteris” group, based on the abovementioned technical scheme. The ROLP isolate was further classified in the aster yellows subgroup (*Candidatus* Phytoplasma sp. AY 16S-group, AY-sg) phytoplasma [8].

Typically, mature rice plants infected with ROLP showed moderate stunting and the appearance of a golden or orange leaf coloration that initiates at the tip and then progresses downward, followed by an inward rolling of leaves and eventually leading to leaf senescence [1,3]. However, the symptoms vary depending on plant conditions and stages, such that the leaves of infected plants show yellow to golden or yellow to deep orange coloration, tiller numbers are slightly to severely reduced, plant height is slightly to severely stunted, and growth habits display erect or spreading phenotypes. Yield is seriously affected due to the poor growth and reduced tillers of plants. At the seedling stage, it is often lethal when the plant is severely infected [1,3]. The insect responsible for its spread is the zigzag-striped leafhopper, *Recilia dorsalis* Motchulsky (*Hemiptera: Cicadellidae*), also known as *Inazuma dorsalis*, and for a long time this species was recognized as the only insect vector that transmits ROLP [2]. Recently however, Li et al. [10] reported that ROLP was also transmitted by another insect species, the green leafhopper (*Nephotettix cincticeps* Uhler) (*Hemiptera: Cicadellidae*) in South China, therefore suggesting much broader infection routes of ROLP in Nature than previously thought.

On the other hand, RYDP reported in rice-growing countries of East and Southeast Asia [11,12] causes general chlorosis of leaves and severely stunted profuse tillers, which are distinct symptoms from ROLD [3]. RYDP is transmitted by green leafhopper species, including *N. virescens* Distant, *N. cincticeps,* and *N. nigropictus* Stål (*Hemiptera: Cicadellidae*) [3,11], which also transmit rice dwarf virus that causes rice dwarf disease (RDD) [13], as well as the rice tungro disease (RTD)-associated viruses, namely rice tungro bacilliform virus (RTBV) and rice tungro spherical virus (RTSV) [14,15]. Symptoms of RDD are also distinct from ROLD and its occurrence in the Philippines is confined only in Midsayap, North Cotabato [13], but it has also been reported in Japan, China, and Korea [16,17,18] RTD is reported to occur in Asian tropics and *N. virescens* is the most efficient vector of the tungro disease [19].

Importantly, it is known that RTD-associated tungro viruses develop symptoms similar to those caused by ROLP infection in rice, such as golden-or orange-colored leaves. Therefore, it often causes confusion at a field level, especially when the rolled senescent leaves have not yet manifested. This might lead to misdiagnosis of ROLD and RTD in many rice-cultivating countries and might be a reason for few reports of ROLD in the early 21st century. In the past decade, however, ROLD has become a reemerging rice disease as it caused field devastation affecting rice yields in India [9], Vietnam [20], Thailand [21], and China [10].

Since the last domestic report of ROLP in 1987 [2], there have been no reports concerning severe damage caused by ROLD in the Philippines. In 2017 and 2019, however, rice plants with yellowing symptoms similar to those of RTD and ROLD were observed at the experimental paddies of the International Rice Research Institutes (IRRI) in Laguna and Mindanao provinces, Philippines. Moreover, the occurrence of *N. virescens* is higher in these rice fields than the ROLP vector—*R. dorsalis*. In this study, the occurrence of ROLP in paddies in the Philippines and possible transmission of ROLP by an alternative insect species were investigated. The results shed light on the increasing importance of ROLD in Southeast and East Asia.

## 2. Results

### 2.1. ROLD Prevalence and Field Symptoms

**Figure 1:**
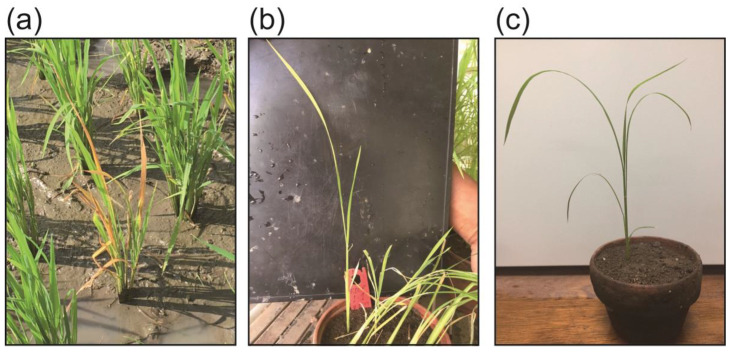
Rice plants showing typical symptoms caused by rice orange leaf phytoplasma (ROLP). (**a**) Typical rice orange leaf disease (ROLD) symptoms appeared on a rice plant at an early tillering stage in a field in Laguna province. (**b**) and (**c**) A rice plant (cv. TN1) in its seedling stage 2 weeks after artificial insect-mediated transmission of ROLP (**b**) and healthy control plants treated with ROLP-negative insects (**c**).

**Figure 2:**
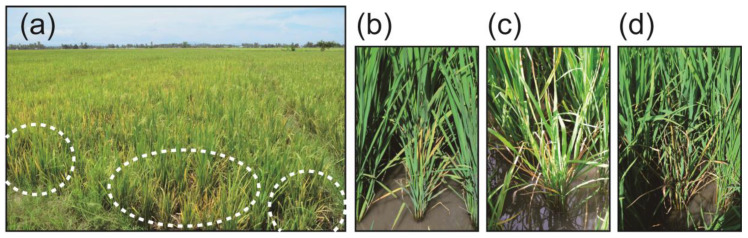
Rice plants infected with ROLP showing varying symptoms at different growing stages. (**a**) The ROLP-infected plants with yellowing symptoms that are sporadically distributed in the field. (**b**) A rice plant with severe stunting, reduced tillers, and orange-colored leaves. (**c**) A plant with golden to deep orange leaf discoloration and inward leaf rolling, which are typical of ROLP-mediated symptoms. (**d**) A heavily diseased plant with severe stunting, reduced tillers, and dried leaves or drying leaves.

Based on field observation, a portion of rice plants in Laguna showed the typical aforementioned ROLD symptoms (Figure 1a). Likewise, in Mindanao, rice plants showing ROLD-like symptoms were sporadically distributed in the rice fields (Figure 2a), while some exhibited tungro-like symptoms that were difficult to distinguish. Indeed, ROLP symptoms varied depending on the fields cultivated, variety, and age of the plant, as previously mentioned. In this field survey, all the ROLD-suspected rice plants, particularly in Mindanao showed a wide range of symptoms, which include a rice plant with severe stunting, reduced tillers, and orange-colored leaves (Figure 2b); a plant with golden to deep orange leaf discoloration and an inward leaf rolling, which are typical orange leaf disease symptoms (Figure 2c); and a plant with severe stunting, reduced tillers, and drying of leaves (Figure 2d).

By nested PCR, 81.7% (49 out of 60) collected in 2017 and 94.7% (36 out of 38 samples) collected in 2019 in Laguna and Mindanao, respectively, were ROLP-positive (Table 1). Thus, most ROLD-suspected rice plants observed in these provinces are considered to have shown symptoms due to the ROLP-infection. Because some of these symptoms are similar to those of RTD, enzyme-linked immunosorbent assay (ELISA) for RTBV and RTSV were conducted. The results showed that no infection of RTBV and RTSV (Supplementary Table S1), thus it is highly expected that the ROLP induced several symptoms in the paddies.

### 2.2. Transmission of ROLP by N. virescens

**Figure 3:**
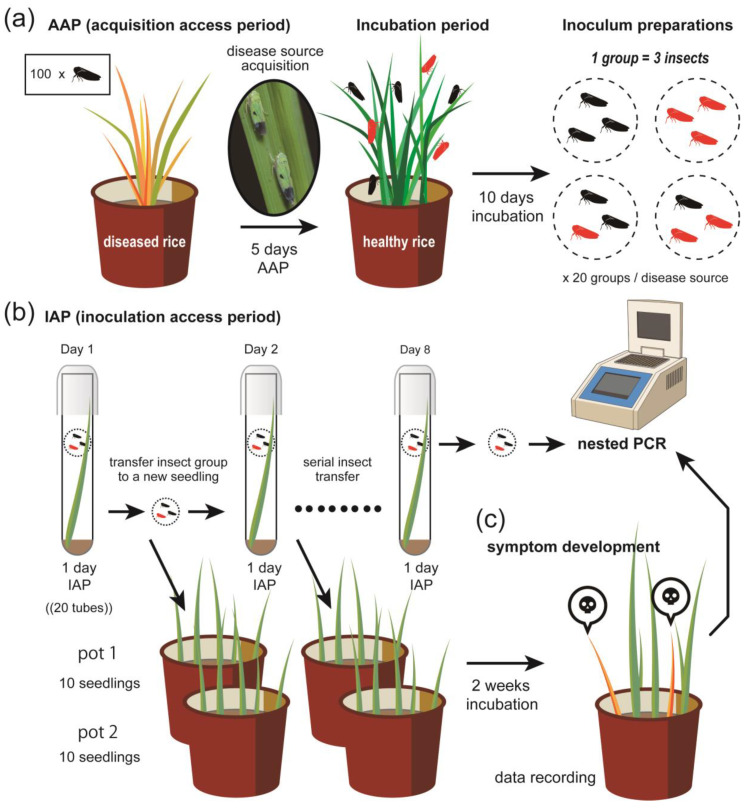
Schematic of the transmission test workflow for ROLP. (**a**) An ROLP-infected rice plant is exposed to 100 virus-free *N. virescens* for a 5-days acquisition access period (AAP), and the insects are reared continuously in a healthy rice plant for 10 days. These possibly viruliferous (red insect symbol) and non-viruliferous (black insect symbol) populations are collected and made into a group of three insects for the transmission test. Twenty insect groups are examined for the single ROLP-source plant. (**b**) A group of insects is confined in a test tube, which contains a 7-day-old rice seedling, and allowed for transmission for 1-day inoculation access period (IAP). Then, this group of insects is transferred to a new test tube with healthy seedling for 1-day IAP. This process is repeated up to 8-days IAP, and insects are collected for ROLP-detection by PCR. (**c**) Inoculated seedlings in (**b**) are transplanted into a pot (10 seedlings per pot) after each 1-day IAP, and grown for 14 days in a greenhouse. The phenotype of each seedling is recorded and leaf samples are harvested for PCR.

Since there is no record of *N. cincticeps* occurrence in the Philippines, *N. virescens* was suspected to be an alternative vector of ROLP. The 60 groups of three insects (20 groups per ROLP-source) were examined for their acquisition of the agent using three ROLP-infected plants (S-1, S-2, and S-9: described as ROLP or disease source) and for the transmission ability to young rice seedlings (Figure 3). After all acquisitions, inoculations, and periods for symptom development, live seedlings were taken into account, while dead seedlings were excluded (maximum at 160 tested seedlings per disease source) to evaluate the transmission based on symptom observation. Infected Taichung Native 1 (TN1) seedlings from the transmission test were slightly stunted and showed leaves with discolored and poor growth (Figure 1b). As a result, 10.6% seedlings (49 out of 464) exhibited symptoms after two weeks from the completion of inoculation access period (IAP) (Table 2).

After 15 days from the initiation of the ROLP acquisition (5-days AAP to 10-days serial IAP), insects were harvested and analyzed for the maintenance of ROLP in their bodies. Nested PCR revealed that 45.0% (27 out of 60 groups of three insects) of the tested insect groups were positive for ROLP, suggesting that almost half of the groups have acquired and maintained the pathogen for at least a week by 5-days acquisition access period (AAP) (Table 2). Note that this detection rate varied between disease-source plants (13, nine, and five groups per 20 groups, respectively). Moreover, not all ROLP-positive insect groups induced symptoms on inoculated seedlings: 74.1% (20 out of 27) showed symptoms, while 25.9% (seven out of 27) showed no symptoms on inoculated seedlings (Table 2). None of the randomly selected asymptomatic leaves from the transmission test were ROLP-positive. Similarly, no symptoms were observed on inoculated seedlings with insects that fed on the control-healthy plant source in the AAP (Figure 1c), therefore, ROLP-detection was negative in insects and seedlings.

### 2.3. Trend Analysis of ROLP Transmission by N. virescens

Looking at detailed transmission trends in the test using the S-1 disease source, which showed the highest transmission score (Table 2), some characteristics of *N. virescens*-mediated ROLP-transfer were observed (Table 3). For instance, insects could transmit ROLP at a high rate in the first and second days after acquisition (DAA) (7/20 seedlings), gradually reduced the rate at 3 and 4 DAA (5/20 and 4/20 seedlings), and almost lose transmissibility at 5, 6, 7 (one/20 seedlings, respectively), and 8 DAA (0 seedlings), suggesting a gradual loss of ROLP-transmissibility over time. Most transfers were continuous for 2–3 days, but some appeared opportunistic (pot 1-No. 8, pot 2-No. 3, and pot 2-No. 5). The insect group of pot 1-No. 3 exceptionally showed continuous transmission (5 seedlings/8 days), whereas three insect groups could not transfer the pathogen even though these insects were expected to carry ROLP (Table 3, gray highlight). These trends were also obvious in the test using S-2 and S-9 disease sources (Supplementary Tables S2 and S3).

### 2.4. Confirmation of ROLP Transmission by 16S rDNA Sequencing

**Figure 4:**
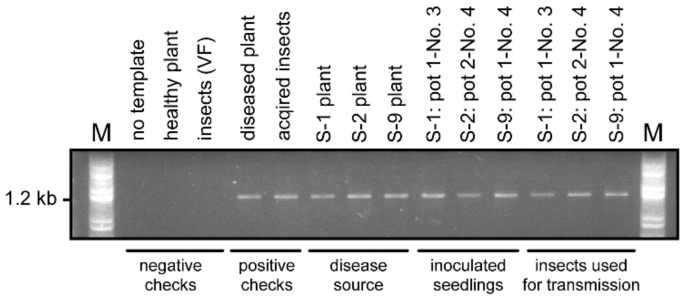
ROLP-detection of rice plants and insects in the transmission test by nested PCR. Two rounds of PCRs using universal primers, P1/P7 and R16F2n/R16R2, were sequentially performed and final products were examined in 1% agarose gel electrophoresis. M, 1 kbp DNA ladder marker.

Three sets of 16S rDNA sequences derived from three ROLP-infected source plants (S-1, S-2, and S-9), three symptomatic seedlings (one per source plant in the transmission test), and three pooled insects used for the transmission test were analyzed (Figure 4). The results showed that all sequenced samples in the replicates and among the groups were completely identical to each other. 

The basic local alignment search tool (BLAST) search with the consensus sequence as a query revealed that the 16S rDNA sequence shared more than 99.0% identity with those of reported ROLP isolates, namely, Coimbatore (IN_Coi2) in India [9], RPKB1-4 in Thailand [21], LD 1 in China [22], and ROL in the Philippines [8] with the Genbank accession numbers, JX290547, KP136894, MIEP01000000, and AB052870, respectively. Also, the 16S rRNA gene sequence (accession number LC586936) obtained from the ROLP-positive plants and *N. virescens* were subjected to iPhyClassifier analysis 30, and the results revealed that the sequence shared 99.9% similarity with that of the “*Candidatus* Phytoplasma asteris” reference strain (GenBank accession: M30790). The virtual restriction fragment length polymorphism pattern [23] derived from the query 16S rDNA F2n/R2 fragment was identical (similarity coefficient 1.00) to the reference pattern of the 16Sr group I, subgroup B (GenBank accession: AP006628). Thus, all ROLP sequences obtained in the Philippines so far belong to the group 16SrI-B. Altogether, our results confirmed that *N. virescens* was able to recover ROLP from the disease source and transmit it into the rice seedlings at relatively low frequency.

## 3. Discussion

In the Philippines, ROLD is currently not recognized as a problematic rice disease, but in 2017 and 2019, yellow-to orange-colored leaf symptoms similar to RTD became noticeable in several rice fields. Indeed, nested PCR and serological tests revealed that 86% (85 from 98 samples) of those yellowing plants in the fields were ROLP-positive, thus ruling out RTV infection (Table 1). Considering the scattering reports of reemerging ROLP from South Asia to East Asia, an understanding of the geographical spread and its mechanism is highly desired for appropriate disease control.

A question arose as to why the occurrence of ROLD-like symptoms was high and yet *R*. *dorsalis*, the sole reported vector of ROLD [2,3,10], was rare in the field, while *N. virescens* was abundant in the investigated fields (Table 1). We revealed that a 45% average of the insects became viruliferous after exposure to diseased plants, and those in part could transmit ROLP either continuously or intermittently to healthy seedlings (Table 2 and Table 3). In a previous report, *R. dorsalis* transmits the ROLD agent persistently at a transmission rate of 13% [2], which is similar to *N. virescens* with a transmission rate of 10.6% (5.1–17.8%) (Table 2). Thus, we concluded that the recent reemergence of ROLP in the Philippines is associated with the new insect vector, *N. virescens*.

Alternatively, *N. cincticeps* has been recognized as a dominant leafhopper in the investigated fields in South China, and its ROLP-transmission rate was 16.7–55.3% in independent transmission tests using field-collected insects that were similar to those by *R. dorsalis* (31.7–61.6%). However, there is no record for ROLP transmission by *N. virescens* due to its poor occurrence [10]. In India, *R. dorsalis* was the carrier of ROLP but other leafhoppers, including *N. virescens* were not [24]. The population of *N. virescens* in South China is low or localized but it is abundant and spread widely in the Philippines and India [25]. *N. cincticeps* is common in temperate countries where low temperature is required for their survival, but there are no substantial records of its occurrence in the Philippines [3]. This geographical distribution of insects might correlate with the nature of ROLP occurrence and transmission. Although it is not well understood why neither *N. virescens* nor *N. cincticeps* have not been revealed to transmit ROLP in previous reports long before, a possible driving factor is the development of new ROLP strains, which resulted in the transmission by previously unrecognized insect vectors [10]. In this regard, it is worth noting a potential example of onion yellows phytoplasma strains, OY-M and OY-NIM, in which the loss of a replicase gene (*orf3* encoding repBP) in the bacterial plasmid of OY-NIM might cause a deficiency in insect-mediated transmission [26].

The insect transmission by *N. virescens* in this study showed different transmission rates among three disease-source plants (Table 2). Moreover, notably, some groups of insect vector of ROLP were unable to transmit the pathogen to treated rice seedlings (Table 2 and Table 3). This is similar to the findings in sugarcane white leaf phytoplasma (SCWLP); despite successful SCWLP-detection in the insect vector, *Yamatotettix flavovittatus* Matsumura (*Hemiptera: Cicadellidae*), the inoculated plants did not show any disease symptoms [27]. The authors inferred that the incubation period of 8 weeks from IAP may be relatively too short to observe the plant symptoms. In this study, *N. virescens* were given a 16–23 days incubation period (5-days AAP, 10-days incubation, and 1-to 8-days IAP) and rice plants were given uniformly 15 days (1-day IAP and 14-days incubation), which coincides with the reported incubation time in *R. dorsalis* (15–33 days) and in rice plant (10–28 days) [2]. Conversely, in India, ROLP was undetected in *N. virescens* collected in the phytoplasma infected rice fields [24]. Failures in the transmission and detection of ROLP might correlate with the phytoplasma accumulation levels in either the source plants or insect bodies, and with relatively short incubation periods in the insect and plants; these are yet to be elucidated in detail.

We have noticed substantial differences in symptom inductions by ROLP between those observed in this study and those in previous reports. For example, all infected TN1 seedlings obtained from serial transmission by *N. virescens* showed yellowing of leaves, stunting, and poor growth (Figure 1b). However, the short, ragged and twisted leaves symptoms reported previously by Hibino et al. [2] were not observed in ROLP-infected plants from the *N. virescens*-mediated transmission test. Furthermore, the diseased fields observed in this study exhibited non-typical ROLD symptoms, instead, some aspects resembled the RTD (Figure 2a–d). Symptom development may be influenced by several factors, such as the rice variety and age of plant tissues when infected with ROLP. Duan et al. [28] observed varying symptoms on eight rice varieties that were inoculated with ROLP by *R. dorsalis*. Symptoms in IR36, IR50, and FK135 included orange discoloration and inward leaf rolling, typical of orange leaves. Symptoms in IR8, IR20, and TN1 were initially similar to those caused by rice ragged stunt virus. The Japonica varieties Reiho and Fukumasari showed symptoms similar to those caused by rice grassy stunt virus. IR20, IR50, and TN1 showed severe ROLP infection than IR8, IR36, and FK135. These findings supported the different field symptoms and transmission rates observed in this study. Furthermore, Wongkaew [29] similarly indicated that the degree of infection severity and symptom variation of SCWLP depends on soil fertility, temperature, cane sette quality, cultural practices, and the amount of phloem-colonized phytoplasma. These factors may also be considered for further studies to clarify the symptom variations and transmission efficiencies of ROLP observed in the field.

The spread of phytoplasma-associated diseases is largely connected to insect vector transmission in the ecosystem. For example, Flavescence dorée phytoplasma (FDP) (16SrV-C and-D) is mainly transmitted in grapevines by American grapevine leafhopper, *Scaphoideus titanus* Ball (*Hemiptera: Cicadellidae*), and causes grapevine yellows disease. However, the monitoring of insect species in vineyards and surroundings revealed new potential insect vectors, such as *Orientus ishidae* Matsumura (*Hemiptera: Cicadellidae*) [30,31]. More recently, a reasonable FDP disperse scenario in Europe was illustrated by Malembic-Maher et al. [32]: briefly, pre-existing asymptomatic FDPs in forest environments moving in and out between natural plant hosts via several insect species that have low efficacy of the pathogen transmission to grapevines, and the introduction of non-autochthonous *S. titanus* from North America resulted in the serious Flavescence dorée epidemics in Europe. In this regard, ROLP was found to naturally infect maize (*Zea mays*) and grassy weeds (*Paspalum paspaloides* and *Eleusine indica*) in China [10] and was confirmed to be vectored by the alternative insect species in this study, suggesting the presence of complex ecological cycle of ROLP in paddies and surroundings that may be varied based on the phytoplasma genotypes.

Altogether, this study observed the variations in the ROLP symptoms and thoroughly investigated the rate of ROLP transmission by *N. virescens*, also it suggests that ROLP variants or strains may have existed in Nature, thereby leading to its transmission by a new vector, *N. virescens*. The high possibility of ROLP incidence in the province of Mindanao, Philippines, where rice is cultivated in large areas of land, is tantamount to post a threat in rice production. Likewise, the varying symptoms of those ROLP-positive rice plants in the field should be further defined for reliable diagnosis and for developing effective control strategies. Consequently, to precisely understand the threat of ROLD in Asian countries, its pathogenicity, mechanism of infection/transmission, global occurrence, and epidemiology should be further investigated.

## 4. Materials and Methods

### 4.1. Plant Source and Leaf Sample Collection

In Laguna province, 60 whole plant samples suspected to be infected with ROLP were collected in 2017, and individual plants were transplanted in pots and kept in a greenhouse. Locations of the field surveys and rice variety information are summarized in Table 1. The second youngest symptomatic leaves from individual plants were sampled and kept at −20 °C. In 2019, symptomatic leaves from 38 plants were collected in several rice fields in Mindanao provinces and were kept at −20 °C. Ninety-eight leaves were tested for ROLP and RTD-associated viruses. The plants that were singly infected by ROLP were used as disease sources for testing ROLP transmission by *N. virescens*.

### 4.2. DNA Sample Preparation

Total DNA was extracted from 1.0 mg symptomatic leaf tissues and leafhoppers using the cetyltrimethylammonium bromide (CTAB) method [33]. The concentration of total DNA was measured using a NanoDrop 1000 Spectrophotometer (Nanodrop Technologies, Inc., Wilmington, DE, USA). Final DNA solutions were adjusted to 50 ng/μL using sterile distilled water and used in subsequent experiments.

### 4.3. Detection of Rice Tungro Viruses

The presence of RTBV and RTSV in plants was determined using ELISA with the RTBV-and RTSV-specific antibodies as previously described by [34]. Briefly, ten-fold diluted extracts (extraction buffer consisted of 0.1 M phosphate buffer, pH 7.0, containing 0.15 M NaCl, 0.05% Tween 20, and 1% NaN_3_) of the second youngest leaves were used for ELISA with specific antibodies of both viruses. A sample with ELISA reading with OD_405_ values > 0.1 was considered to be infected [35].

### 4.4. Detection of ROLP

ROLP was detected from leaves and insects using nested PCR. For the first-round amplification, 1.0 μL DNA (50 ng/μL) was used as a template with primers P1 and P7 [5], followed by the second-round amplification using primers R16F2n and R16R2 with a 0.5 μL DNA template from the first-round PCR [6]. The program for the first-round PCR was set to 94 °C for 2 min; followed by 30 cycles at 94 °C for 30 s, 52 °C for 30 s, and 72 °C for 2 min; and concluding with 72 °C for 10 min. The program for the second-round PCR was similar to that described above, except that the annealing temperature was set to 50 °C. The first-and second-rounds PCRs produced 1.4 and 1.2 kbp fragments, respectively. The final products amplified by nested PCR were run in 1.0% agarose gel electrophoresis prestained using GelRed (Biotium Inc., Fremont, CA, USA). The gels were examined under Bio-Rad gel documentation system information (Bio-Rad, Hercules, CA, USA).

### 4.5. ROLP Insect Transmission Test

Three infected plants (S-1, S-2, and S-9) collected in Laguna, which were identified as ROLP-positive by PCR were used as ROLP sources for acquisition by newly emerged adult green leafhoppers, *N. virescens*. Pre-germinated TN1, a rice variety susceptible to ROLP, was sown in a pot and grown for 7 days in the greenhouse. Approximately 100 virus-free (healthy), newly emerged adult *N. virescens* reared in the greenhouse were given a 5-day AAP to each ROLD-source plants and then continuously confined to a healthy susceptible TN1 plant for another 10 days to complete the minimum incubation period of the pathogen in the insect [2,9] (Figure 3a). The individual 7-day-old healthy rice seedling was transferred to a test tube (one seedling/tube) and a group of three potentially viruliferous insects was confined per test tube and covered with caps. The insects were forced to feed on seedling for 24 h of IAP. After 1 day of forced inoculation, the insects were serially transferred to another set of healthy seedlings consecutively for up to 8 days (Figure 3b). After each day of IAP, inoculated seedlings were transplanted in pots at a rate of 10 seedlings per pot (2 pots/ROLD source, for each IAP) (Figure 3c). Then, on the last day of inoculation, insects were retrieved and kept at −20 °C. Similarly, another set of newly emerged adult virus-free green leafhoppers given AAP in the healthy rice plant were used as control. All inoculated seedlings were kept in the greenhouse for symptom development. Visual symptoms assessment was performed after 2 weeks, which is the minimum pathogen incubation period in the plant [2] (Figure 3c). All plants with symptoms were recorded and symptomatic leaves were sampled and tested for the presence of ROLP and RTVs as described above. Similarly, the remaining insects stored at −20 °C were tested for the presence of ROLP using nested PCR following the protocol described above. Due to quarantine restrictions, only the ROLP-source plants from Laguna province were used for the transmission test. The experimental flow is visually summarized in Figure 3.

### 4.6. Sequence Analyses

All nested PCR products from ROLD-source plants used in the transmission test, including the symptomatic seedlings and their corresponding insects used for inoculation were sent to Macrogen (Seoul, South Korea) or Apical Scientific Sdn Bhd (Selangor, Malaysia) for nucleotide sequence determination. All generated sequences derived from the 16S rDNA fragment with the same pair of primers of each plant and insect sample were aligned using ClustalW multiple alignments program and consensus sequences were created using BioEdit sequence alignment editor Version 7.2.5 [36]. To confirm their phytoplasmal origins, each of the consensus sequences was subjected to a BLAST available online at https://blast.ncbi.nlm.nih.gov/Blast.cgi. Further taxonomic confirmation was made using a phytoplasma species demarcating software, iPhyClassifier analysis 30 [37], available at http://plantpathology.ba.ars.usda.gov/cgi-bin/resource/iphyclassifier.cgi.

## Figures and Tables

**Table 1:** Rice orange leaf phytoplasma (ROLP) detection from leaf samples collected in the paddies in the Philippines.

Year	Province	Municipality	Barangay ^a^	Rice Variety	Detection ^b^
2017	Laguna (IRRI), Luzon	Los Baños	College	unknown	49/60
2019	Davao del Sur, Mindanao	Hagonoy	Sinayawan	Nsic Rc 286	8/8
	Davao del Sur, Mindanao	Matanao	New Murcia	Nsic Rc 224	2/2
	CompostelaValley, Mindanao	Compostela	Tamia	Nsic Rc 160	7/9
	Davaodel Norte, Mindanao	Sto Tomas	Kinamayan	unknown	9/9
	Davao Oriental, Mindanao	Banaybanay	Mobongcogon	Nsic Rc 160	3/3
	Davao Oriental, Mindanao	Banaybanay	Cabangcalan	unknown	7/7

^a^ The smallest category of political jurisdiction in the Philippines. ^b^ Number of ROLP-positive plants per number of total plants, which were examined using PCR.

**Table 2:** Transmission of ROLP to susceptible TN1 rice seedlings by the leafhopper, *Nephotettix virescens.*

Disease Source Plants	ROLP-Detection (Rice Seedlings) ^a^	ROLP-Detection (Insect Groups) ^b^	Symptom Development by ROLP-Positive Insect Groups	
			(+) ^c^	(−) ^d^
S-1	27/153	13/20	10	3
S-2	14/155	9/20	5	4
S-9	8/157	5/20	5	0
H-control ^e^	0/149	0/20	NA ^f^	NA ^f^

^a^ Number of ROLP-detected rice seedlings per number of total plants tested, which were examined using PCR. ^b^ Number of ROLP-detected insect groups per number of total groups tested, which were examined using PCR. ^c^ Number of ROLP-positive insect groups that showed symptoms on treated plants. ^d^ Number of ROLP-positive insect groups with no symptom development on treated plants. ^e^ Healthy plant used for acquisition access to insects. ^f^ Not applicable.

**Table 3:** Breakdown of the result of ROLP-transmission by *N. virescens* using the disease source S-1, and the PCR-detection of ROLP in treated insect groups.

Disease Source	Pot No.	Insect Group No.	Days of Serial Inoculation ^a^								No. Insect Left	ROLP in Insect ^c^
			D1 ^b^	D2	D3	D4	D5	D6	D7	D8		
S-1	1	1	− ^d^	−	−	−	−	−	−	−	3	−
		2	−	−	−	−	−	−	−	−	3	−
		3	+ ^d^	+	+	+	−	+	−	d	2	+
		4	−	−	−	−	−	d	−	d	1	+
		5	+	−	−	−	−	−	−	d	2	−
		6	−	x	−	d ^d^	−	−	−	−	2	−
		7	−	+	+	+	−	−	−	−	3	+
		8	+	−	−	+	−	−	−	−	3	+
		9	x ^d^	−	−	−	−	−	−	d	2	+
		10	x	−	−	−	−	−	−	−	3	−
	2	1	−	−	x	−	−	−	−	d	2	+
		2	−	−	−	−	−	−	−	−	3	−
		3	+	−	+	−	−	−	−	−	3	+
		4	−	−	x	−	−	−	−	−	3	−
		5	+	−	+	−	−	−	+	−	3	+
		6	+	+	−	+	−	−	−	−	3	+
		7	+	+	x	−	+	−	−	−	3	+
		8	+	+	+	−	−	−	−	−	3	+
		9	−	+	x	−	−	−	−	d	2	+
		10	−	+	−	−	−	d	−	d	2	+

^a^ Insects were given acquisition access period of 5 days and continuously confined to healthy seedlings for 10 days before sequential 1 day-inoculation to individual 7-day-old TN1 seedling. ^b^ Day 1–8: daily transfer of insect groups to healthy TN1 seedlings. ^c^ ROLP was detected using nested PCR. Highlighted frames are inoculated seedlings that did not show symptoms but detected ROLP in insects. ^d^ Symbols: x, death of a seedling; +, inoculated seedlings with symptoms; −, asymptomatic seedlings; d, death of an insect.

## Supplementary Materials

The following are available online at https://www.mdpi.com/2076-0817/9/12/990/s1, Table S1: Enzyme-linked immunosorbent assays (ELISA) for rice tungro bacilliform virus (RTBV) and rice tungro spherical virus (RTSV) from leaf samples collected in the paddies in the Philippines, Table S2: Breakdown of the result of Rice Orange Leaf Phytoplasma (ROLP)-transmission by *Nephotettix virescens* using the disease source S-2, and the PCR-detection of ROLP in treated insect groups, Table S3: Breakdown of the result of ROLP-transmission by *N. virescens* using the disease source S-9, and the PCR-detection of ROLP in treated insect groups.
