# Supplementary material for: Reemerging Rice Orange Leaf Phytoplasma with Varying Symptoms Expressions and Its Transmission by a New Leafhopper Vector—Nephotettix virescens Distant
Source: Pathogens. 2020 Nov 26;9(12):990. doi: 10.3390/pathogens9120990 (PMC7761223; doi:10.3390/pathogens9120990)
Supplement: Supplementary file 1 [file pathogens-09-00990-s001.pdf]

Supplementary Table S1. Enzyme-linked immunosorbent assays (ELISA) for rice tungro bacilliform virus (RTBV) and rice tungro spherical virus (RTSV) from leaf samples collected in the paddies in the Philippines [a]

Year	Province	Municipality	Barangay [b]	Antibody against [c]	
				RTBV	RTSV
2017	Laguna (IRRI), Luzon	Los Baños	College	0/60	0/60
2019	Davao del Sur, Mindanao	Hagonoy	Sinayawan	0/8	0/8
	Davao del Sur, Mindanao	Matanao	New Murcia	0/2	0/2
	CompostelaValley, Mindanao	Compostela	Tamia	0/9	0/9
	Davaodel Norte, Mindanao	Sto Tomas	Kinamayan	0/9	0/9
	Davao Oriental, Mindanao	Banaybanay	Mobongcogon	0/3	0/3
	Davao Oriental, Mindanao	Banaybanay	Cabangcalan	0/7	0/7

[a] All collected leaf samples used for phytoplasma detection were also examined by ELISA with the RTSV- and RTBV-specific antibody.

[b] The smallest category of political jurisdiction in the Philippines.

[c] No. infected plants / total samples tested.

Supplementary Table S2. Breakdown of the result of Rice Orange Leaf Phytoplasma (ROLP)-transmission by *Nephotettix virescens* using the disease source S-2, and the PCR-detection of ROLP in treated insect groups.

Disease source	Pot No.	Insect group No.	Days of serial inoculation [a]								No. Insect left	ROLP in insect [c]
			D1 [b]	D2	D3	D4	D5	D6	D7	D8		
S-2	1	1	- [d]	-	-	-	-	-	-	-	3	-
		2	-	-	-	-	-	-	-	-	3	+
		3	-	-	-	-	-	-	-	-	3	+
		4	-	-	+	+	-	+	-	-	3	+
		5	-	-	-	-	-	-	-	-	3	-
		6	-	-	-	-	-	-	-	-	3	-
		7	**x** [d]	+ [d]	-	-	-	-	-	+	3	+
		8	-	-	-	-	-	-	-	**d** [d]	2	-
		9	-	**x**	-	-	**x**	-	-	-	3	-
		10	-	-	-	-	-	-	-	-	3	+
	2	1	-	+	+	+	+	-	-	**d**	2	+
		2	-	-	+	-	+	-	-	-	3	+
		3	-	-	-	**x**	-	-	-	-	3	-
		4	-	-	+	+	-	-	-	+	3	+
		5	-	-	-	**x**	-	-	-	-	3	+
		6	-	-	-	-	-	-	-	-	3	-
		7	-	-	-	-	-	-	-	-	3	-
		8	-	-	-	-	-	-	-	**d**	2	-
		9	-	-	-	-	-	-	-	-	3	-
		10	-	-	-	-	-	-	-	-	3	-

[a] Insects were given acquisition access period of 5 days and continuously confined to healthy seedlings for 10 days before sequential 1day-inoculation to individual 7-day-old Taichung 1 (TN1) seedling.
[b] Day 1–8: daily transfer of insect groups to healthy TN1 seedlings.
[c] ROLP was detected using nested PCR. Highlighted frames are inoculated seedlings that did not show symptoms but detected ROLP in insects.
[d] Symbols: x, death of a seedling; +, inoculated seedlings with symptoms; -, asymptomatic seedlings; d, death of an insect.

Supplementary Table S3. Breakdown of the result of ROLP-transmission by *N. virescens* using the disease source S-9, and the PCR-detection of ROLP in treated insect groups.

Disease source	Pot No.	Insect group No.	Days of serial inoculation [a]								No. Insect left	ROLP in insect [c]
			D1 [b]	D2	D3	D4	D5	D6	D7	D8		
S-9	1	1	- [d]	-	-	-	-	-	-	-	3	-
		2	-	-	-	-	-	-	-	-	3	-
		3	-	-	-	-	-	-	-	**d** [d]	2	-
		4	+ [d]	+	+	-	+	-	-	-	3	+
		5	-	-	-	-	-	-	-	**d**	2	-
		6	-	-	-	-	-	-	-	-	3	-
		7	-	-	-	-	-	-	+	-	2	+
		8	-	-	-	-	-	-	-	**d**	2	-
		9	**x** [d]	-	-	-	-	-	-	-	3	-
		10	-	-	-	-	-	-	-	-	3	-
	2	1	-	-	-	-	-	-	-	**d**	2	-
		2	-	-	-	-	-	-	-	-	3	-
		3	-	-	**x**	-	-	-	-	**d**	2	-
		4	-	-	-	-	-	-	-	-	3	-
		5	-	-	-	-	-	-	-	-	3	-
		6	-	+	-	-	-	-	-	+	3	+
		7	-	-	-	-	-	-	-	**d**	2	-
		8	-	-	-	-	-	-	-	**d**	2	-
		9	-	+	-	-	-	-	-	-	3	+
		10	-	**x**	-	-	-	-	-	+	3	+

[a] Insects were given acquisition access period of 5 days and continuously confined to healthy seedlings for 10 days before sequential 1day-inoculation to individual 7-day-old TN1 seedling.
[b] Day 1–8: daily transfer of insect groups to healthy TN1 seedlings.
[c] ROLP was detected using nested PCR.
[d] Symbols: x, death of a seedling; +, inoculated seedlings with symptoms; -, asymptomatic seedlings; d, death of an insect.
